# Beyond 8-methoxypsoralen as the photosensitizer for extracorporeal photopheresis

**DOI:** 10.3389/fonc.2022.996973

**Published:** 2022-10-18

**Authors:** Yandy Marx Castillo-Aleman

**Affiliations:** Department of Immunology, Abu Dhabi Stem Cells Center (ADSCC), Abu Dhabi, United Arab Emirates

**Keywords:** 5-aminolevulinic acid, 5-methoxypsoralen, 8-methoxypsoralen, apoptosis, extracorporeal photopheresis, photoimmunology, photosensitizer, phototherapy

## Introduction

Since the United States Food and Drug Administration (FDA) approved the use of extracorporeal photopheresis (ECP) in the palliative treatment of cutaneous T-cell lymphoma (CTCL) in 1988 (1), many other indications have been successfully explored. However, most of these approaches still demand further randomized clinical trials, including graft-vs.-host disease (GvHD), rejection of solid organ transplantation, and a wide range of autoimmune diseases.

ECP is a leukapheresis-based therapy where the patient’s whole blood is collected and separated into its different components; the collected leukocytes are treated extracorporeally with a photosensitizing agent and then exposed to ultraviolet-A (UV-A) irradiation before reinfusion.

Interestingly, despite the vast marketing experience of the ECP therapy gathered over more than 30 years and the manufacturing improvement of photopheresis devices, no significant changes have occurred around the photosensitizing drugs, which remain restricted to 8-methoxypsoralen (8-MOP, methoxsalen).

Few preclinical and early phase clinical trials have explored different photosensitizers during the last decade. Other ECP photosensitizing agents with equivalent (or better) safety, efficacy, and cost profiles could be available in the marketing landscape of low-income regions that cannot afford the current financial and economic costs of these promising therapies.

This Opinion paper aims to raise some regulatory considerations regarding the conventional photosensitizing agent (8-MOP), emphasizing the potential usefulness of other drugs and non-pharmacological systems for photosensitization during ECP.

## ECP regulatory landscape

The FDA considers a photopheresis system a “combination product” comprising two regulated components: a drug (photosensitizer) and a device (leukapheresis machine) (2). Furthermore, the European Medicines Agency (EMA) refines the regulation of medicinal products used with a medical device by defining “integral,” “co-packaged,” and “referenced” combinations. Those referenced products “combine two (or more) product information of the medicinal product refers to a specific medical device to be used, and the specified medical device is obtained separately by the user of the medicinal product” (3).

For instance, 8-MOP branded as UVADEX^®^ Sterile Solution (Mallinckrodt Pharmaceuticals, Ireland- United States) has been granted FDA authorization in combination with one specific photopheresis device only (UVAR^®^, and its more recent superseding device, THERAKOS^®^ CELLEX^®^, both Therakos Inc., Mallinckrodt Pharmaceuticals) (4) that correspond to closed, “online” ECP methods. In Europe, ECP products manufactured using open “offline” methods are subject to Advanced Therapeutic Medical Product (ATMP) regulations (5).

Nevertheless, it is widely accepted that a combination product’s primary mode of action (PMOA) is “the single mode of action expected to make the most significant contribution to the overall intended therapeutic effects of the combination” (2). In ECP, the PMOA relies on photosensitizers, comprised of drugs and irradiation systems, rather than the leukapheresis device. A few online and integrated systems for cellular collection, photoactivation, and infusion are available (e.g., THERAKOS^®^ CELLEX^®^ device and AMICUS^®^ Blue ECP system, Fresenius Kabi, Germany); however, evidence also shows that ECP has effectiveness using different marketed photosensitizers and photoactivation devices (e.g., MacoGenic G2 irradiation device, Macopharma, France). Those offline platforms treat leukocytes harvested by other apheresis machines not necessarily intended for ECP protocols (e.g., SPECTRA OPTIA^®^ Apheresis System, Terumo BCT, Japan) and are not approved as combination products, as mentioned earlier.

## Discussion

### Irradiation devices for ECP

Photoactivation devices play pivotal roles in ECP PMOA. The UV-A irradiation dose (intensity and duration) and the plate film thickness can polarize to either the immunizing or tolerogenic effects exerted by ECP (6, 7). These dual capabilities are crucial for clinical conditions that can be treated with photopheresis: while enhanced immune responses are desirable in cancer scenarios (e.g., CTCL as the primary approved indication), ECP tolerizing effects are required in cases such as solid organ transplantation, GvHD, and autoimmune diseases.

### Photosensitizer drugs for ECP: Capabilities beyond 8-MOP

Unfortunately, there is no consensus as to what in-process and pharmaceutical controls should be performed for ECP products; therefore, there is a critical need for harmonized quality-control assays, as stated and summarized as (5):

- Cellular composition of ECP products;
- Induction of apoptosis in lymphocytes;
- Monocyte polarization;
- Psoralen photoadducts;
- T cell suppression; and,
- Capacity for antigen cross-presentation.

Although many questions about PMOA of photosensitizers and ECP as a whole procedure remain inconclusive, the main advances in pharmacological development, manufacturing, quality controls, and preclinical and clinical uses, correspond to 8-MOP.

Like other psoralens, 8-MOP, upon UV-A photoactivation (wavelength: 315˗400 nm), conjugates and forms covalent bonds with DNA that lead to monofunctional (addition to a single strand) and bifunctional (crosslinking of psoralen to both DNA strands) photoadducts. Lastly, it results in inhibition of DNA synthesis and cell division (4, 8). The apoptotic fate of extracorporeally exposed cells consists of the ECP PMOA generating antigen-specific immune and clinical responses, which are not described here (8).

Traditionally, the combined administration of psoralens (orally or topically) with UV-A (PUVA) has been widely used to manage psoriasis and CTCL patients. Eventually, non-8-MOP psoralens and other photosensitizer drugs, such as those used in photodynamic therapy (PDT), can be applied in ECP (9). **Table 1** summarizes distinctive characteristics, preclinical, and clinical evidence of current and potential photosensitizers for ECP.

**Table 1:** Current and potential photosensitizer drugs for ECP.

Photosensitizer	ATC code	Chemical structure	Current indications	Required light (wavelength, nm)	ECP regulatory status	Preclinical ^(P)^ and clinical ^(C)^ evidence on ECP
**Psoralens for systemic use**						
8-methoxypsoralen (8-MOP, methoxsalen)	D05BA02	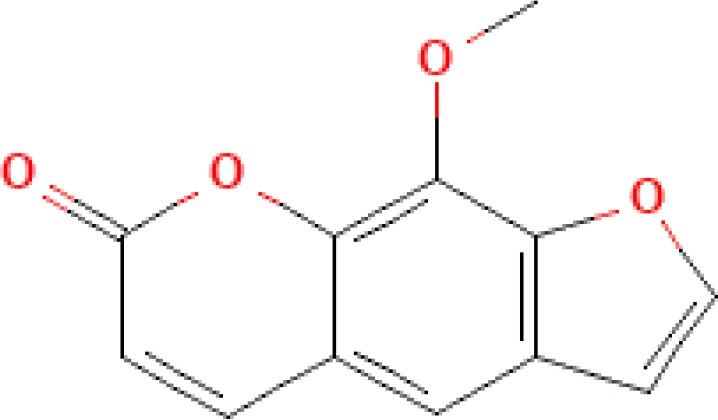	- Psoriasis - Vitiligo - CTCL (ECP)	UV-A (315˗400)	- Marketing authorization (CTCL)	- Vast post-authorization experience ^(C)^ - Ongoing clinical trials for new indications (e.g., NCT05168384, NCT05413005) ^(C)^
Trioxsalen (trimethylpsoralen, trisoralen)	D05BA01	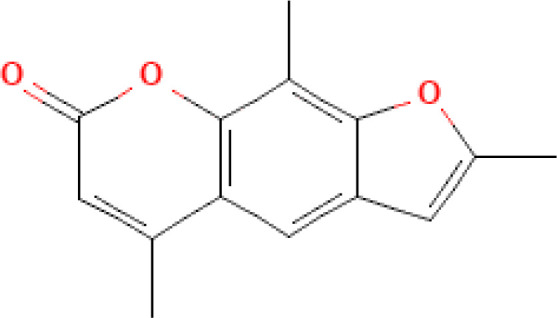	- Vitiligo (discontinued)	UV-A (315˗400)	- Unknown or not reported	- Unknown or not reported
5-methoxypsoralen (5-MOP, bergapten)	D05BA03	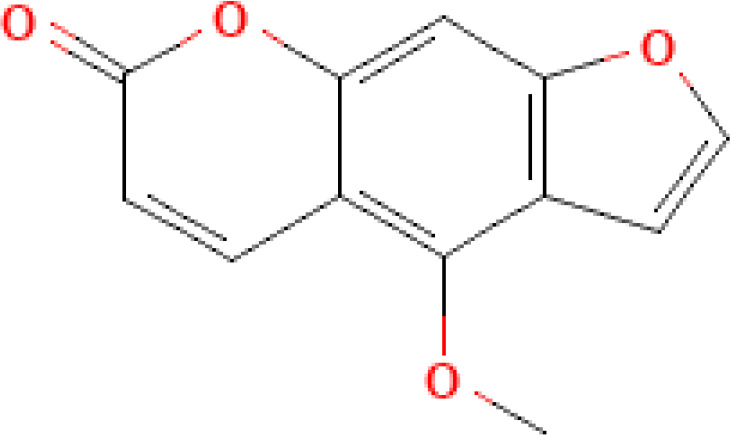	- Psoriasis - Vitiligo - CTCL	UV-A (315˗400)	- Unknown or not reported	- (10) ^(C)^ *
**Sensitizer used in PDT ^§^**						
5-aminolevulinic acid (5-ALA)	L01XD04	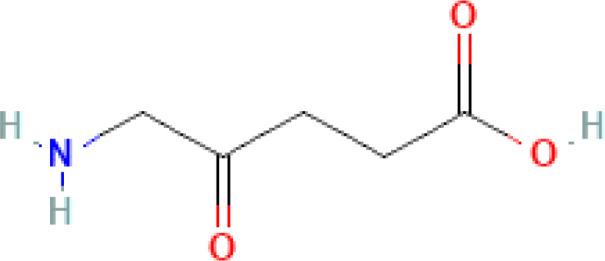	- Actinic keratosis - Glioma (intraoperative optical imaging agent)	Visible light (400˗635)	- Preclinical studies - Early phase clinical trials	- (11) ^(P)^ ** - (12) ^(P)^ - (13) ^(P)^ - (14) ^(P)^ - (9) ^(C)^ - NCT04164849 ^(C)^
**Electromagnetic spectrum of light applied for ECP**			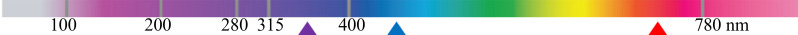			

*Case report on concomitant use of 5-MOP (PUVA) and 8-MOP (ECP); **In vitro administration of hexaminolevulinate, an ester of 5-ALA

**^§^** Other drugs in this group include: porfimer sodium, methyl aminolevulinate, temoporfin, efaproxiral, padeliporfin (unknown or not reported use for ECP)

ATC, Anatomical Therapeutic Chemical; CTCL, Cutaneous T-Cell Lymphoma; ECP, Extracorporeal Photopheresi; PDT, Photodynamic therapy; PUVA, Psoralen and UV-A; UV-A, Ultraviolet-A Light (Source: PubMed, PubChem, and ClinicalTrials.gov databases).

In 1996, Wolf et al. described the successful treatment of a patient with photoaccentuated erythroderma and idiopathic CD4 T lymphocytopenia using 5-methoxypsoralen (5-MOP)-PUVA, and ECP with 8-MOP concomitantly (10). However, there are no publications of ECP procedures delivered with 5-MOP or trioxsalen (trimethylpsoralen) to date.

In contrast, PDT photosensitizers, such as 5-aminolevulinic acid (5-ALA), have emerged in preclinical (11–14) and early phase clinical trials (9) as an alternative to the authorized 8-MOP for ECP. PDT typically involves systemic or topical administration of a lesion-localizing photosensitizer (e.g., 5-ALA) and its subsequent activation by visible light (400˗780 nm, represented by blue and red arrowheads in the electromagnetic spectrum of **Table 1**), primarily resulting in a singlet oxygen-induced photodamage in the exposed cells (12, 14). Nevertheless, further preclinical research is needed to clarify ECP mechanisms of action, resulting in appropriate harmonized quality controls for these photosensitizers.

The alternative use of 5-ALA is supported by *ex vivo* investigations that show that this photosensitizer affects T-cells from chronic GvHD patients more selectively and efficiently than those treated with 8-MOP-ECP, through the formation of protoporphyrin IX. Consequently, reducing the number of ECP treatments can be achieved using 5-ALA (9). This finding occurs even with a UV-A light source resembling emission spectral wavelengths to those of the built-in certified UV-A commercial photopheresis systems (340˗410 nm) (12, 14).

The clinical applications of novel photosensitizing drugs are limited based on the number of published clinical trials, procedures, and patients enrolled. For instance, the cited work of Christensen et al. reported 82 ECP treatments with 5-ALA in five chronic GvHD patients who responded poorly to 8-MOP-photopheresis after a minimum of three months of treatment. Although safety and tolerability were found to be adequate (9), no additional clinical evidence is yet available. Only another phase I/II clinical trial using 5-ALA-ECP has been registered on ClinicalTrials.gov (NCT04164849) for patients with active Crohn’s disease, as shown in **Table 1**. Still, more clinical studies are required to demonstrate differences in safety, efficacy, and cost profiles compared to the approved 8-MOP.

### Apoptosis induced by light: Are ECP-photosensitizers still needed?

Insulting agents that trigger “immediate” pre-programmed cell death (pre-PCD) apoptosis include PDT, UV-A1 (340˗400 nm), and agents that generate singlet-oxygen damage to mitochondrial membranes (e.g., with 5-ALA). “Intermediate” apoptosis occurs to a significant extent within 4 h, but requires more than 30 min, and high doses of either UV-B (290˗320 nm) or UV-C (200˗290 nm) radiation, and any agent that activates a membrane receptor containing a death domain, such as Fas/CD95/APO-1. “Delayed” apoptosis occurs well after 4 h (or days), and examples of agents that induce primarily delayed PCD apoptosis are UV-B, UV-C, X-rays, and any agent that causes significant DNA damage (15).

However, different wavelength photons have been used to treat various diseases without the need for photosensitizing drugs, resulting in necrosis but also inducing cell apoptosis (desired PMOA of ECP), with consequent changes in the production of soluble mediators (cytokine profiles), modulation of the expression of cell-surface associated molecules, and damage in pathogenetically relevant cells (cytotoxicities) (15, 16). It has been demonstrated that *in vitro* UV-A1 and UV-B irradiation alone induced T cell apoptosis, which reduces inflammatory infiltrates in T cell-mediated skin diseases (16). Still, the chances to use other types of radiations to induce apoptosis extracorporeally require further photoimmunologic studies.

## Conclusions

Additional developments in manufacturing and evaluating innovative ECP-photosensitizing drugs are rising needs that should accompany the development of photopheresis medical devices. Moreover, alternative non-pharmacological systems for *in vitro* photosensitization and apoptosis induction are strategies that should be further explored. Compliance with regulatory requirements derived from the emerging knowledge might extend the new indications for ECP, benefiting more patients and healthcare systems.

## Author contributions

YMCA is the sole author and agrees to be accountable for the content of the work.

## Acknowledgments

The author gratefully acknowledges Eng. Diana Patricia Hermida Vitar, MSc, for critical reading of the manuscript.

## Conflict of interest

The author is an Investigator and Principal Investigator of two clinical trials examples of new indications of ECP using 8-MOP (NCT05168384 and NCT05413005, respectively; **Table 1**).

## Publisher’s note

All claims expressed in this article are solely those of the authors and do not necessarily represent those of their affiliated organizations, or those of the publisher, the editors and the reviewers. Any product that may be evaluated in this article, or claim that may be made by its manufacturer, is not guaranteed or endorsed by the publisher.
